# Rethinking Social Cognition in Light of Psychosis: Reciprocal Implications for Cognition and Psychopathology

**DOI:** 10.1177/2167702616677079

**Published:** 2017-02-10

**Authors:** Vaughan Bell, Kathryn L. Mills, Gemma Modinos, Sam Wilkinson

**Affiliations:** 1 Division of Psychiatry, University College London; 2 Institute of Cognitive Neuroscience, University College London; 3 Department of Psychosis Studies, Institute of Psychiatry, Psychology and Neuroscience, King’s College London; 4 Department of Philosophy, Durham University

**Keywords:** psychosis, social cognition, delusion, hallucination, schizophrenia

## Abstract

The positive symptoms of psychosis largely involve the experience of illusory social actors, and yet our current measures of social cognition, at best, only weakly predict their presence. We review evidence to suggest that the range of current approaches in social cognition is not sufficient to explain the fundamentally social nature of these experiences. We argue that social agent representation is an important organizing principle for understanding social cognition and that alterations in social agent representation may be a factor in the formation of delusions and hallucination in psychosis. We evaluate the feasibility of this approach in light of clinical and nonclinical studies, developmental research, cognitive anthropology, and comparative psychology. We conclude with recommendations for empirical testing of specific hypotheses and how studies of social cognition could more fully capture the extent of social reasoning and experience in both psychosis and more prosaic mental states.

Cognitive studies of psychosis present us with a paradox: the positive symptoms of psychosis largely involve illusory social experiences, and yet our current measures of social cognition, at best, only weakly predict the presence of positive symptoms and more strongly predict negative symptoms (Mehta et al., 2013; Ventura, Wood, & Hellemann, 2013; Ventura, Wood, Jimenez, & Hellemann, 2013).

One probable reason is that our current concepts of social cognition do not capture the full range of relevant cognitive function for disturbed social experience in psychosis (Gallagher & Varga, 2015; Schilbach, 2016; Yager & Ehmann, 2006). We argue here that a core feature of the phenomenology of psychosis is the experience of illusory social agents and that social agent representation is a useful framework for understanding disturbed social cognition in psychosis, as well as social reasoning and interaction more generally. This article reviews the evidence from studies of psychosis to support this position and subsequently discusses the feasibility of a social agent representation approach in terms of the evidence from studies of normal social cognition.

First, it is worth clarifying what we have in mind when discussing social agent representation. Building on previous work on auditory hallucinations (Bell, 2013; Wilkinson & Bell, 2016), we argue that human social agent representation involves the ability to create, use, and maintain internal representations of social actors for use in both implicit and explicit social cognitive function. In particular, social agent representation (a) is present to differing degrees of complexity throughout development; (b) involves the capacity to internalize models of social actors including their physical and psychological attributes and requires these representations to be maintained and updated through implicit and explicit learning; (c) can involve representations of differing specificity and complexity—from individualized to general, from sparse to rich; (d) is used in both “online” and “offline” social reasoning to predict behavior (i.e., during live social interaction and when the agents being considered are absent); and (e) could be drawn on for reasoning about described, notional, or hypothetical individuals as needed.

We suggest that psychosis is a model example of disordered social agent representation, and we begin by reviewing the research on the experience of anomalous social agents in psychosis and note how current theories of psychosis do not adequately address this core aspect of the condition. With regard to typical cognition, the role of social agent representations has been subject to research across a number of disciplines and domains, but this evidence has not been well integrated. Subsequently, we bring this research together to note how a social agent representation approach is supported by research on normal and comparative social cognition and note links with studies of psychosis where relevant. We conclude with specific hypotheses generated by this approach and discuss recommendations for future research in terms of understanding psychosis and more prosaic social cognitive functioning.

## Illusory Social Phenomenology in Psychosis

One of the most striking aspects of the delusions and hallucinations of psychosis is that they are, in large part, social experiences, by which we mean that they most commonly involve the experience of being affected by illusory social actors, as opposed to being largely, or equally, about natural or nonintentional events. Similarly, we can contrast the social experience of psychosis with the experiences of people diagnosed with, for example, social phobia, where the concerns principally center on the thoughts and actions of genuine social actors. Psychosis is distinguished by the experience of having vivid but illusory social agents intrude and often persist in your social world.

These delusions or hallucinatory experiences may “involve” illusory social agents of numerous types. For example, they may involve personally identified agents (for example, a family member or a historical or religious figure), groups (neighbors or the CIA), supernatural or fictional figures (angels or TV characters), or social agents who are entirely idiosyncratic and seem to be recognized solely by the individual who experiences them. There are many striking examples in the literature: James Tilly Matthew’s belief that he was being persecuted by a gang of magnetic spies using an “air loom,” each of whom he identified individually (Haslam, 1810/1988; Jay, 2012); Mellor’s (1970) report of a patient who experienced television host Eamonn Andrews inserting thoughts into his mind; and Jackson, Hayward, and Cooke’s (2011) case series including “Lucy,” who heard the voices of a “guardian angel, grandparents, and dead relatives,” to name but a few. These illustrative examples are supported by strong evidence from systematic studies, which we review in the next section, that show that psychotic symptoms commonly have clear social content and involve the experience of illusory social agents.

### Auditory verbal hallucinations

Among psychotic symptoms, auditory verbal hallucinations are the most widely researched in terms of social content (review in Bell, 2013). Hallucinated voices can differ in the extent to which they are experienced as representing specific agents, from voices that do not seem to represent clearly defined agents (such as shouts or murmurs from crowds) to voices that are experienced as being individual agents to voices that reflect specific individuals (Wilkinson & Bell, 2016) with the latter being the most common. In a large psychiatric sample of 199 voice hearers, McCarthy-Jones et al. (2014) reported that 70% of voice hearers had voices that were identifiably similar to people they had encountered in the past. Nayani and David (1996) reported that 61% of voice hearers from a psychiatric sample could ascribe a specific identity to their voices and another 15% experienced voices that were described as familiar but unknown. In a qualitative study of 50 psychiatric voice hearers, Beavan (2011) reported that characterizing voice identity was a central theme of the experience of hallucinated voices, present in all of the study participants. A more recent qualitative study by Upthegrove et al. (2016) on hallucinated voices in young people with first-episode psychosis found that the concept of “entity, as though from a living being with complex social interchange” was one of the two main categories of characteristics (the other being differing feelings of being in control of the voice). Corstens and Langdon’s (2013) phenomenological study on 100 psychiatric voice hearers reported that “the most prevalent reported experience was to hear voices that could be clearly personified in terms of age, gender, and name.” In their study, 94% of people reported adult voices and 47% reported that they had the experience of a voice representing a family member and 47% a known acquaintance. Woods, Jones, Alderson-Day, Callard, and Fernyhough (2015) reported on a study of 153 voice hearers, of whom 69% experienced voices that were characterful in some way—that is, were experienced as “people or person-like entities with distinct characteristics such as gender, age, patterned emotional responses or intentions.”

Further studies have reported that the majority of voice hearers engage with their voices through interactive conversations (Garrett & Silva, 2003; Leudar, Thomas, McNally, & Glinski, 1997) and that voice hearers report social relationships with hallucinated voices that are experienced and understood in similar ways to relationships with people (Hayward, Berry, & Ashton, 2011). In the perceived relationship with the voice, the power dynamics reflect power dynamics in external social relationships, and this is a significant mediator of distress (Paulik, 2012). This evidence suggests that hallucinated voices are typically interacted with and experienced as psychologically credible social agents.

Nonsocial auditory hallucinations, which may include mechanical sounds like bells or whistles, or may include voice hallucinations that are not experienced as representing or coming from social agents—such as palinacousis (the experience of illusory echo on genuinely heard phrases)—can occur on their own or alongside socially themed hallucinations (Woods et al., 2015). However, these nonsocial experiences are clearly in the minority, of which the majority are social and agentive in nature (Wilkinson & Bell, 2016).

### Delusions

Delusions also have a strongly social component, as has been reported by studies on delusional theme. Persecutory delusions are consistently reported as the most common type (Ellersgaard et al., 2014; Junginger, Barker, & Coe, 1992; Musalek, Berner, & Katsching, 1989; Yamada, Nakajima, & Noguchi, 1998), which are by definition social, as are delusional jealousy, erotomanic delusions, and most types of delusional misidentification. Other delusions may not be social *by definition* but commonly are. For example, grandiose delusions may commonly involve beliefs about social links to prominent people (Suhail & Cochrane, 2002), whereas delusions of external control and passivity, although not defined in relation to social actors, are invariably social, with individuals believing that they are being controlled or influenced by external groups or individuals (Hirjak, Breyer, Thomann, & Fuchs, 2013; Spence, 2001). A study by Green et al. (2006) on the content of persecutory delusions in psychiatric patients that specifically investigated the presence and types of social agents found in the delusions reported that they typically involved single (50%) or multiple (50%) persecutors that were human in nature (81.2%) and identifiable to the individual (53.6%). When the social content of delusions from psychiatric patients with psychosis is studied broadly, the vast majority involve experiences of specific social agents (Bell et al., in preparation).

To contrast, a nonsocial psychotic symptom would be one where the experience involves no other social actors. For example, delusions of negation (i.e., Cotard’s delusion) or infestation are often nonsocial in this manner (for example, the cases in Solla, Cannas, Orofino, & Marrosu, 2015; Stanciu, Penders, & Oxentine, 2015). Delusions solely about the natural world (“there will be a tsunami”; Palmira, Stompe, Narbekovas, & Bunevicius, 2008) can also be nonsocial if they are not elaborated to include other social actors. However, as with hallucinations, delusions that lack social content are in the minority.

### Social phenomenology and current theories of psychosis

Despite the strong social theme of these experiences, most theories of delusion or hallucination formation do not address why these phenomena are typically social, rather than nonsocial. Waters et al.’s (2012) integrated model of cognitive mechanisms in auditory verbal hallucinations focuses almost entirely on explaining how internally generated mental phenomena could be experienced as nonself and does not attempt a cognitive explanation of why auditory verbal hallucinations are typically experienced as social agents rather than just hallucinated but depersonalized words or speech (the content of auditory verbal hallucinations is described as possibly “determined by factors such as perceptual expectations, mental imagery, and prior experience/knowledge [e.g. memories],” but no further elaboration is given). In other words, this model, like most others, accounts for why the experience is “not me” but offers no account of why it is of “somebody else.” Similarly, Allen, Larøi, McGuire, and Aleman’s (2008) and Allen et al.’s (2012) structural and functional brain connectivity model does not address social aspects of hallucinated voices despite significant overlap between the identified areas involved in auditory verbal hallucinations and the “social brain” (Kennedy & Adolphs, 2012).

Current theories of delusions are similarly inadequate in explaining the prominence of illusory social agents in delusions. Some theories exclude social factors (Braun & Suffren, 2011; Frith, 1992; Hemsley, 1993), whereas others tackle social factors implicitly by restricting themselves to persecutory delusions that are by definition social but do not explain why most delusions are typically social rather than nonsocial (Bentall, Corcoran, Howard, Blackwood, & Kinderman, 2001; Freeman, 2007; Freeman & Garety, 2014). Two-factor accounts of delusions (reviewed in Coltheart, Langdon, & McKay, 2011) implicitly suggest that the first factor, an anomalous experience, could be social in content if it involved social perception—such as reduced autonomic responding to familiar people in Capgras delusion—but does not explain why this is usually interpreted in terms of illusory social agents (in the case of Capgras, “identical looking impostors” having replaced family members) rather than a delusional but alternative explanation without illusory agents (“my relatives have become empty shells”) except to say that this happens due to “reasoning deficits” in the second factor. Kapur’s (2003) aberrant salience model also does not explain why social experiences are more common, as presumably aberrant salience would affect the perception of social and nonsocial experiences equally. Indeed, the fact that social isolation increases the risk of psychosis and anomalous experiences (Broome et al., 2005) suggests that a simple model where socially themed psychotic symptoms are more likely to arise due to aberrant salience occurring during the most commonly encountered (e.g., social) experiences would also be unlikely. Nelson, Whitford, Lavoie, and Sass (2014) do suggest a brief explanation for why psychotic symptoms are typically social in theme, hypothesizing that psychotic symptoms arise due to the weakening of the influence of memory-based context on current interpretations and that these representations are commonly social in nature, although they provide no evidence for the claim. Clearly, there is a wide explanatory gap between the social phenomenology of psychosis and the current scope of theories that attempt to explain its symptoms.

### Psychosis as a window on social agent representation

This contrast between the phenomenology and current scientific accounts suggests that the common presence of illusory agents in psychosis is worthy of explanation but is currently underinvestigated. We argue that the most parsimonious explanation for the presence of illusory agents in psychosis is that, rather than emerging de novo from a breakdown in other cognitive processes, they reflect dysfunction of existing social cognitive systems for social agent representation. Here, we draw on the conceptual tools of cognitive neuropsychiatry, where symptoms are understood as a breakdown in the function of normal cognition and may be a guide to its structure (Halligan & David, 2001), to suggest that the social phenomenology of psychosis may arise from a dysfunction in social agent representation—an important organizing principle for normal social cognition.

## A Place for Agent Representation in Social Cognition

Traditional approaches to social cognition have been understood as being built on a set of neuropsychological functions that, using Schilbach’s (2014) distinction between online and offline social cognition, typically reflect “online” or “live” social interaction to allow people to make decisions during the interaction period. These have included agency detection, to distinguish agentive objects from nonagentive objects (Johnson, 2003), person and affect recognition via face (Todorov, Ida, Evans, & Haxby, 2007), and voice perception (Belin, Bestelmeyer, Latinus, & Watson, 2011) and mental state attribution—traditionally conceptualized as “mindreading,” “mentalization,” or “theory of mind” (Frith & Frith, 2012; Olsson & Ochsner, 2008). Notably, this latter aspect is typically conceptualized in the literature as having conscious metacognitive components as well as implicit and automatic aspects (Mitchell & Phillips, 2015), and we note that the implicit functions would be characterized as an “online” function as we describe it here, whereas the conscious metacognitive aspect could be either an “online” function or equally applied to “offline” social cognition (as discussed in the following section). However, it is clear from the extent of current social cognition research that there is an inclination towards understanding processes used in “live” or “online” social interaction.

### Offline social agent representation

Although these are clearly essential functions, this approach neglects the fact that everyday social cognition is frequently, and perhaps most commonly, focused on individuals who are not present and therefore does not involve direct perception and reasoning about ongoing social communicative acts, which is what is most typically tested in lab studies. There are exceptions, such as theory of mind problem-solving tasks, but these are limited in terms of the potential extent of “offline” social cognition as they typically require reasoning about hypothetical “one-off” or brief scenarios that do not require models of social agents to be maintained over time, tracked, or substantially updated as they would be in our most common forms of social interaction. Evidence for this primarily “offline” nature of social cognition has been found in several studies. Studies that have analyzed the content of social speech suggest that the majority of our talk is about people not present at the conversation (Dunbar, 1993, 2004; Dunbar, Marriott, & Duncan, 1997). In terms of mental state attributions, Bryant, Coffey, Povinelli, and Pruett’s (2013) experience sampling study found that these were made more frequently when people were not interacting with others than during social interaction itself. Mar, Mason, and Litvack’s (2012) experience sampling study of daydream content found that the majority of individuals (73.2%) reported that their internal thoughts “frequently” or “always” involved other people. Even when considering inner speech, which is typically conceptualized as self-generated, self-directed conscious thought, evidence suggests that even here social cognition is a prominent factor. McCarthy-Jones and Fernyhough (2011) reported that one quarter (25%) of all “inner speech” reported by the participants involved representations of other people’s voices.

From a parasocial perspective, it is also worth noting that we can easily apply “offline” social reasoning to individuals with whom no “live” social interaction will likely ever take place (for example, in the case of politicians or celebrities) and, indeed, for “individuals” who only exist as social concepts because they are deceased, are fictional characters in novels or films, or purely exist as social stereotypes (for example, we can consider how a “policeman” or a “grandmother” might react in a certain situation, despite not having a specific personal identity in mind). Indeed, research on the use of “theory of mind” in understanding fiction (e.g., Dodell-Feder, Lincoln, Coulson, & Hooker, 2013; Panero et al., 2016) is entirely based on this premise, and we note that much theory of mind research focuses on, for want of a better word, “fictional” characters in experimental paradigms where the participants are aware that the judgments they make are not about real people or genuine social situations.

We suggest that these “offline” social cognitive processes may be more influential in the content of psychotic experiences than “online” social cognition, given that symptoms more commonly reflect this type of social experience, even when colored by perceptual distortions. For example, the situation of hearing a hallucinated voice is more akin to imagining a conversation, even when colored by perceptual experience, than it is like having a face-to-face conversation, which is affected by a range of nonauditory cues (e.g., Ross et al., 2011; Tjan, Chao, & Bernstein, 2014). Indeed, physical auditory properties in hallucinated voices are less prevalent than social agentive properties (Woods et al., 2015), suggesting the social cognitive dimension has been overlooked by largely perceptual theories. Along similar lines, delusions typically involve a belief that illusory social actors are “behind” or “involved” in events, even when the events or communicated acts being misinterpreted are genuine (Startup & Startup, 2005), suggesting that social cognition is key even without a clear hallucinatory basis for the experience.

### A developmental perspective on social agent representation

Fields (2014) has noted that the social agent construct appears to be a “fundamental, and quite possibly innate, ontological category for human infants.” The ability to distinguish between animate and inanimate objects develops in early infancy, with infants associating some animate properties with people by 6 months of age (Rakison & Poulin-Dubois, 2001). Infants as young as 5 months old will attribute goals to agentive (self-propelled) nonhuman objects (Luo & Baillargeon, 2005). Indeed, agent-based reasoning seems pervasive throughout early childhood. Kelemen (2004) has reviewed evidence to show that infants approach natural phenomena with a “promiscuous teleology”—that is, a tendency to primarily understand objects as having being made for a purpose by or as intentional agents. This is despite the fact that Western parents tend to give and reinforce explanations for objects that involve nonintentional causal mechanisms or events rather than intentional ones, suggesting that “agent-first” reasoning can be present despite cultural indoctrination to the contrary (Kelemen, Callanan, Casler, & Pérez-Granados, 2005).

By their first birthday, infants are not only able to associate mental attributes to specific agents (Buresh & Woodward, 2007), but they can also track the identities of individual social actors (Xu & Carey, 1996). These building blocks of social agent representation remain throughout life, providing general and sparse level representations that are later complemented by the eventual availability of more specific and socially detailed representations as cognition progresses throughout development.

Studies in adults show that biological motion is detected and processed differently to similarly complex nonbiological motion (Blake & Shiffrar, 2007; Neri, Morrone, & Burr, 1998). Indeed, judgments of intentionality are readily inferred from relatively simple motion contingency (Blakemore, Sarfati, Bazin, & Decety, 2003; Santos, David, Bente, & Vogeley, 2008). In psychosis, these judgments are affected in that people with paranoid delusions tend to overattribute intentionality (Blakemore et al., 2003) and communicative intentions (Okruszek et al., 2015) from agentive motion, suggesting an overinterpretation of agentive intention from implicit agent-detection processing.

In middle childhood, from approximately 4 to 12 years old, explicit reasoning about social agents emerges and develops (mostly studied as “theory of mind”; Wellman, Cross, & Watson, 2001) as does the experience of illusory social agents. In a large sample of children between the ages of 5 and 12 years, approximately half reported having interacted with imaginary companions (Pearson et al., 2001), and evidence suggests that children are able to integrate physical characteristics and personality into their models of imaginary companions with “whom” they have conversations (Gleason, Sebanc, & Hartup 2000). It is worth noting that the presence of imaginary companions has been found to be associated with both the tendency to hear words amid a sound stimulus of unstructured phonemes (Fernyhough, Bland, Meins, & Coltheart, 2007) and better social cognitive development in terms of representing and understanding others’ mental states (e.g., Davis, Meins, & Fernyhough, 2014; Roby & Kidd, 2008) even in children at high risk for developing problem behaviors (Taylor, Hulette, & Dishion, 2010). In contrast, imaginary companions associated with negative psychiatric outcomes are typically not experienced as under voluntary control (“noncompliant imaginary companions”; Jardri et al., 2014) or remain beyond their typical developmental period in middle childhood, suggesting an altered developmental pathway that affects social agent representation.

In adolescence, there is now a significant amount of evidence that the development of identity is partly built on the perspectives of others and that tracking and modeling the perspectives of specific individuals develops markedly during this period as an essential ability for navigating this challenge (Blakemore & Mills, 2014; Pfeifer & Peake, 2012; Sebastian, Burnett, & Blakemore, 2008). Notably, although hallucinations are common throughout childhood, new onset hallucinated voices predicted very little general psychopathology in 7- to 8-year-olds (Bartels-Velthuis, Jenner, van de Willige, van Os, & Wiersma, 2010) but a 2.5 to 5 fold increase in general psychopathology in 12 to 13-year-olds (Bartels-Velthuis, van de Willige, Jenner, van Os, & Wiersma, 2011), suggesting that the experience of persistent illusory social agents is more likely to be pathological during specific points in social development. Indeed, the developmental trajectory of social agent representation also seems to mirror the trajectory of illusory social agent experiences that include both developmentally normal and psychosis-like experiences.

**Fig. 1.**
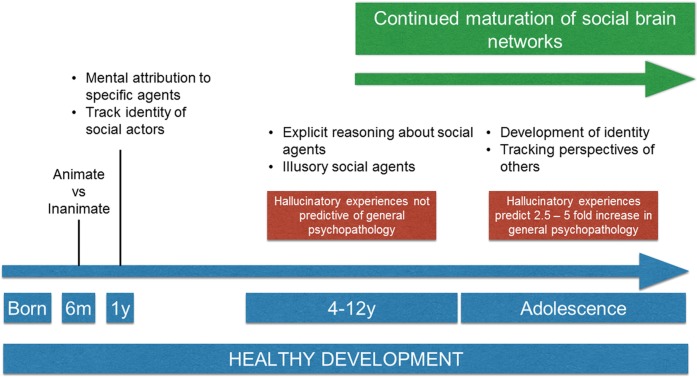
Development of normal and anomalous social agent representation through childhood and adolescence.

### Social agent representation: Evidence from neuroimaging

Research on recognition of individuals has long posited a notional “person identification node” in the cognitive system for face and perception, which has been described as drawing on a semantic memory for recognition (Belin et al., 2011; Bruce & Young, 1986). In terms of more complex information, “person knowledge” has been cited as being retrieved during recognition tasks and has been shown to doubly dissociate from general autobiographical memory after brain injury, with each showing the potential to be independently impaired from the other (Haslam, Kay, & Hanley, 2002).

Imaging studies suggest that people knowledge involves representation distinct from objects and nonhuman animals based on patterns of neural activation. An fMRI study by Mitchell, Heatherton, and Macrae (2002) reported that semantic judgments about people and objects could be distinguished by a unique pattern of brain activity that included the medial prefrontal cortex (mPFC), superior temporal cortex, intraparietal sulcus, and fusiform gyrus. Using a similar paradigm, Mason, Banfield, and Macrae (2004) asked participants to report whether a common set of behaviors could be performed by people or dogs, noting that reasoning about people was particularly associated with activation in the right middle and medial frontal gyri. In a facial recognition task, Todorov et al. (2007) reported that previously associated personal characteristics modulated fMRI activation even when irrelevant to the task, suggesting that person knowledge was being retrieved automatically.

Several studies have tested whether patterns of neural activation can be used to distinguish social agents from the self, or individual social agents from each other. Tomlin et al. (2006) used an economic exchange game and found fMRI activation in the cingulate gyrus was sufficient to discriminate self from other across response types in the task. Other studies have found that self–other distinctions are more apparent in the mPFC, with “dissimilar others” showing less overlap than “similar others” when activation is compared with self-judgments (e.g., Mitchell, Macrae, & Banaji, 2006; Schurz, Kogler, Scherndl, Kronbichler, & Kühberger, 2015).

Studies on “personality models” (Park, 1986; Park, DeKay, & Kraus, 1994) have noted that we seem to maintain and update representations of individuals’ personalities on which we can consider future or notional behavior. An imaging study based on this work by Hassabis et al. (2014) reported that the individual being considered, albeit in an experimental task limited to four notional individuals, could be identified solely through the pattern of activation in the mPFC. This is in line with findings from Welborn and Lieberman (2015), who reported neural evidence for “person-specific theory of mind” in that target individuals could be identified through mPFC activation in “theory of mind” tasks. Interestingly, activation in this area seems to be specific to the perception that the participant is interacting with a human agent, as mPFC activity distinguishes perceived human from perceived nonhuman agents even when behavior is identical (Chaminade et al., 2012) and people representation can be distinguished from objects and locations based on activation patterns (Szpunar, Jacques, Robbins, Wig, & Schacter, 2014).

From the evidence to date, mPFC activity seems sufficient to distinguish between individual social agents, although it is unlikely that the spatial distribution of neural representations will remain so straightforward as evidence for the link between brain activity and social agent representation becomes better understood. Nevertheless, the fact that individual social agents, albeit characterized by experimentally controlled differences, can be distinguished by neural activation suggests that individual agent representation is plausible in terms of differences in the functioning of key areas in the social brain.

### Social agent representation and theory of mind

Theory of mind is, for many, the paradigmatic cognitive ability for representing and understanding other minds. It is worth noting that this term is used to mean a range of different things in the literature, and the research has been noted for its “inconsistent and under-specified uses of relevant terminology” (Mitchell & Phillips, 2015), meaning that it is almost always possible to find a study on theory of mind that seems to cover a particular area of social cognition. To clarify, the concept of theory of mind we are using here is the explicit, largely conscious metacognitive system involving the ability to infer and predict the intentions, thoughts, desires, behaviors, and beliefs of other people (Frith & Frith, 2012; Green et al., 2008). We argue that social agent representation is not simply theory of mind, although theory of mind is clearly an important component. First, it is worth noting that although theory of mind is impaired in people with schizophrenia, the deficit is independent of the presence of the positive symptoms of psychosis, including paranoid delusions (Bora Yücel, & Pantelis, 2009; Garety & Freeman, 2013), which, as noted, largely involve the pathological presence of illusory social agents. There is some evidence that positive symptoms may be linked to overuse of “theory of mind” (“hypermentalizing”; Backasch et al., 2013; Clemmensen et al., 2014; Montag et al., 2011), although this would not be sufficient to explain why illusory social agents tend to feature in positive symptoms rather than just the misattribution of intentions to existing agents. Similarly, theory of mind deficits (for example, after brain injury) are not in themselves predictive of psychotic symptoms. However, we also want to note that social agent representation involves representation and reasoning beyond what is usually considered within the remit of theory of mind (Frith & Frith, 2012; GelmanGelman, Noles, & Stilwell., 2014)—not least the tracking, maintenance, updating, and de novo creation of social agent representations that includes both psychological and physical characteristics.

Indeed, as we note more fully in the following section, the tracking, maintenance, and updating of social agent representations can be done in animals without “theory of mind” as it is normally understood. It is also worth noting that there is evidence that social-agent representation has seemingly automatic and “irresistible” effects on the wider cognitive system in humans. For example, the perceived presence of an observer has a measurable impact on an individual’s task performance even when it is irrelevant to task completion (Capozzi, Cavallo, Furlanetto, & Becchio, 2014; Samson, Apperly, Braithwaite, Andrews, & Bodley Scott, 2010), suggesting an involuntary propensity to co-represent the perspectives of external agents (Gallotti & Frith, 2013).

## Comparative Aspects of Social Agent Representation

### Cross-cultural aspects of anomalous social agents

Boyer (2003) and Bering (2006) note that the tendency to attribute natural events to unseen agents (what Barrett & Johnson, 2003, call hypersensitive agency detection, and what Shermer, 2012, calls agenticity) seems to be a universal characteristic of the world’s cultures. Several researchers have suggested that many of the principal features of religion or belief in supernatural agents rely on core aspects of social cognition that have primarily evolved for dealing with everyday social interaction (Atran & Norenzayan, 2004; Bloom, 2007; Boyer, 2003; Gervais, 2013), suggesting that belief and consideration of supernatural agents emerges from social cognitive processes. Indeed, an fMRI study of Christian religious participants during prayer showed robust activation of social cognitive networks when praying to God (Schjoedt, Stødkilde-Jørgensen, Geertz, & Roepstorff, 2009), and sociological studies have shown how putative spiritual entities are integrated into communities as social agents (Blanes & Espírito Santo, 2014). Notably, belief and experience of spiritually conceptualized supernatural agents seem to be on a continuum with psychosis-like experience, suggesting that similar cognitive systems may underlie their representation (Farias, Underwood, & Claridge, 2013; Pechey & Halligan, 2011; Peters, Day, McKenna, & Orbach, 1999).

### Evolutionary role of social agent representation

One potential objection to citing social agent representation as a core focus of social cognition would be to say that it simply labels a general application of the cognitive system with no specific functional significance. It would be possible to argue, perhaps, that we equally have a “transport representation system” that distinguishes, maintains, and updates representations of specific vehicles. Perhaps a related but more focused criticism would be to suggest that social agent representation is simply an application of other, already defined, higher level functions of the social cognitive system. Apart from the evidence presented above, which suggests that social agent representation seems to be cognitively and neurally distinct from the representation of nonsocial agents, we suggest there are also good evolutionary reasons why the ability to recognize, maintain, and update representations of social agents would be a core organizing principle in social cognition and not simply one of any number of tasks for which it could be used. Working from the most basic level upward: Being able to distinguish agentive from nonagentive threats, being able to distinguish same-species agents from different-species agents, being able to distinguish between same-species agents, being able to maintain and update representations of agents to facilitate social organization and hierarchy, and being able to reflect on and reason about social agents when they are not present or are entirely notional are clearly key survival mechanisms and would likely be heavily selected for.

In terms of psychosis, we note that common delusional themes are often an exaggeration of common survival concerns for social animals (Green & Phillips, 2004; McKay & Dennett, 2009). In light of the fact that this “exaggeration” occurs to the point where illusory agents tend to occur, we suggest that this reflects a dysfunction in a human system that has been subject to selection pressure and shaping during evolution.

### Social agent representation in other species

Similarly, evidence against the fact that social agent representation might simply be a label for a task completed by higher level cognitive or social cognitive functions is the fact that social agent representation is clearly present in animals who do not have higher level social cognition, such as “theory of mind.” For example, Cheney and Seyfarth (1990) have shown that East African vervet monkeys do not have “theory of mind” skills but do have highly complex social structures that require social agent representation and tracking. Indeed, social agent representation is likely to be preserved in a wide range of social animals (Brent, Chang, Gariépy, & Platt, 2014; Emery & Clayton, 2009) with the complexity, rather than the presence of the social agent representation, varying with the cognitive capacity of the animal.

## Conclusions and Implications for Future Research

From the evidence presented here, we argue that social agent representation is a key organizing principle of social cognition that follows a clear developmental pathway, is essential for both minimal survival and maximal social success, has both implicit and explicit components, is key for both online and offline social cognition, is culturally universal, and can be seen to break down in terms of the misrepresentation of social agents in the delusions and hallucinations of psychosis. We argue that social agent-based reasoning needs to be further explored in social cognition research and that insights into social cognition can be gained from better understanding anomalous social agent representation, most notably in psychosis.

It is worth noting that we are not suggesting that this is a new or distinct “component” of social cognition but instead presenting a teleological view that highlights social agent representation as an organizing principle of which many of the already established processes form a part. We are also not suggesting that this is the only or primary involvement of social cognition in psychosis given extensive research on the role of existing social cognition measures in predicting negative symptoms particularly (Green, Horan, & Lee, 2015). However, it is also true that there is clearly a conceptual gap between our current concepts of social cognition and (a) how they explain social cognition as it is used in everyday life and (b) how they explain mental state anomalies in psychosis, and it is possible that a study of social cognition from a social agent representation perspective may lead to the identification of new cognitive mechanisms.

Although there has been a significant amount of research on psychosis and the misattribution of actions to the self or other, which implies but does not specify the role of other agents, the fact that psychosis often involves the experience of specifically characterized “others” suggests that much of this research could be extended from distinguishing self–other to characterizing how the illusory “others” come to dominate social cognition. Clearly, a socially richer approach to social cognition research is needed, in line with Schilbach’s (2016) advocacy for a “second-person neuroscience” that includes genuine social interaction within experimental paradigms and that more heavily emphasizes social interaction rather than social observation in the understanding of psychiatric disorders.

Even with existing interaction or simulated-interaction paradigms, however, one difficulty is that most typically involve the serial or instant judgment of others’ intentions during experimental tasks (usually set up with a specific scenario) and do not involve the need to create, track, or update a representation of a particular social agent beyond a few trials at most. These sorts of limited paradigms are likely to be sensitive to general cognitive or social cognitive impairments but not the social agent representation difficulties most commonly present in psychosis, which involves the experience of illusory but relatively long-lived social agents, rather than the experience of rapidly created spontaneous agent representations.

Studies that require participants to implicitly distinguish and track social agents (for example, by distinguishing individual agents based solely on their behavior in an economic exchange game and using this information to inform future interactions) are likely to have additional ecological validity, in terms of biases on the formation and maintenance of social agents. We also argue that paying more attention to the phenomenology in the form and content of anomalous social agents is likely to provide an important window into normal social cognition, hopefully answering the current paradox as to why current measures of social cognition predict so little of what are fundamentally social symptoms.

Another area suitable for further investigation is social cognition with regards to illusory social agents themselves. Experimental studies typically group participants based on their clinical presentation (or lack of), but very little research has been done on, for example, social judgments regarding the social agents whom a person with psychosis believes, for example, is persecuting him or her or “whom” they experience as auditory hallucinations. Comparing these judgments to judgments about real or imagined social agents may be revealing in terms of differences in social cognitive performance.

A social agent representation approach to social cognition would also raise some specific hypotheses. In developmental terms, the ability to internalize models of social agents that exist in the “real world” should develop over time and should be the basis for being able to use spontaneously created social agent representations in social problem solving. This approach would also predict that although there should be some overlap between social neural networks that support “online” and “offline” social cognition, performance in tasks that test these respective abilities should be differently affected by altering parts of the neural networks that most support them. Similarly, neural activation associated with experiences of anomalous social agents, associated with but not restricted to psychosis, should reflect areas involved in social agent representation in normal social cognition.

One area of interest that has recently arisen is the extent to which internal representations of the self may be related to representations of others with the suggestion that they may be different uses of the same core representation system (Friston & Frith, 2015; an overlap also reflected in the neuroimaging literature discussed previously). Indeed, one of the least understood aspects of psychosis are delusions of identity change, and we might speculate as to whether these are, paradoxically, social in nature, due to the representation of the self relying on some of the same social agent representation mechanisms that are used to represent other agents.

There is clearly a need for a much greater understanding of social agent representation both in normal social cognition and in psychopathology. Although we have focused here on psychosis, as it seems to be the most striking example of anomalous and potentially dysfunction social agent representation, this approach also has clear relevance for other diagnoses where social cognition has been implicated, such as in autism and psychopathy (Happé & Frith, 2014). We hope that both methodological and theoretical innovations will better explore and develop this hypothesis in the future.
